# Information needs as perceived by caregivers and patients following stroke: a qualitative systematic review

**DOI:** 10.1186/1471-2458-12-S2-A36

**Published:** 2012-11-27

**Authors:** Nor Haty Hassan, Syed Mohamed Aljunid, Peter Davis

**Affiliations:** 1 United Nations University-International Institute for Global Health, Universiti Kebangsaan Malaysia Medical Centre, Jalan Yaacob Latiff, 56000 Kuala Lumpur, Malaysia; 2 School of Nursing, Faculty of Medicine and Health Sciences, University of Nottingham, Nottingham, NG7 2HA, United Kingdom

## Background

Despite the availability of information related to stroke, patients and caregivers claimed that they are not receiving adequate information. The aim of this review was to critically appraise selected articles that addressed information needs perceived by caregivers and patients following stroke, in various stages of stroke: from the acute to the community phase.

## Materials and methods

Literature search involved main databases namely EMBASE, MEDLINE, Cumulative Index to Nursing and Allied Health Database (CINAHL), Psych INFO and British Nursing Index (BNI) through library online service. Keywords used were: information, educational, needs, carers, caregivers, family, patients, and stroke. Search attempts also include the use of other terms such as grounded theory, phenomenology, ethnography, case study, with the hope that one of these terms was used in the abstract or title of relevant studies. The systematic literature search covered published papers from the year 1980s to 2009. Manual search were also carried out on selected non-indexed journals and databases, and letters to editor which report research results, or conference abstracts.

## Results

In all, 223 potentially relevant papers were identified. However, only 19 papers met the inclusion criteria. After the assessment of the methodological quality of the research was done (JBI-QARI Critical Appraisal Instrument), only 11 qualitative studies were finally used in this review. Six themes were identified, namely: information needs, strategies, resources, lack of information, barriers and support information. Patients and caregivers information needs would vary according to the stages and severity of stroke. The most required information at each stage of stroke was information relating to illnesses, while physical care skills were perceived as essential to caregivers prior to discharge.

## Conclusions

Findings support the need for information to be individualized, based on priorities and vary over time. This has practical implications in the way information is related to patients and their caregivers.

